# *Brassica rapa* subsp. Chinensis juice enhances *Bacillus subtilis* selectively in leafy green production

**DOI:** 10.1111/1758-2229.13154

**Published:** 2023-03-14

**Authors:** Yingyue Li, Michelle Mei Zhen Ten, Cliff An Ting Tham, Yan Xi Lim, Yuyun Lu, Dan Li

**Affiliations:** ^1^ Department of Food Science and Technology Faculty of Science, National University of Singapore Singapore Singapore

## Abstract

*Bacillus subtilis* (BS) is a well‐known beneficial microorganism for plants but is not competitive in the plant rhizosphere microbiome. We report the selective support of *Brassica rapa* subsp. Chinensis (Xiao Bai Cai) juice (XBCJ) on BS both in hydroponic nutrient solution and the plant rhizosphere of lettuce. After 2 weeks of being inoculated in the lettuce rhizosphere, the *Bacillus* population was enumerated at 3.30 ± 0.07 log CFU/unit in the BS group and at 5.20 ± 0.39 log CFU/unit in the BS + XBCJ group (*p* < 0.05). Accordingly, lettuce crops from the BS + XBCJ group were significantly higher than the control group for all of the tested biomass‐related parameters (*p* < 0.05). The treatment did not significantly affect the texture, colour, moisture contents, total phenolic contents, or antioxidant activities of the lettuce crops (*p* > 0.05). Non‐target ultra‐performance liquid chromatography‐quadrupole time‐of‐flight mass spectrometry (UPLC‐Q‐TOF‐MS) suggested that phenolic compounds could be the key class of phytochemicals being responsible for the selectivity. High‐throughput RNA‐based 16S rRNA gene sequencing and analysis were performed to depict the influence of BS and XBCJ over the global microbiome compositions of plant rhizosphere.

## INTRODUCTION

In the face of climate change, population growth, ecosystem degradation, and increasing resource scarcity, people are dedicating great efforts to establishing a more sustainable agri‐fresh produce supply chain in recent years (Krishnan et al., 2020; Parajuli et al., 2019). Urban farming is one of the possibilities under the spotlight, as it allows a higher yield of crops with the same amount of space compared to traditional farming and avoids unnecessary food miles from the farm to the fork. In an indoor environment, environmental variables, such as temperature, humidity, carbon dioxide level, and lighting are calibrated and thus much better controlled (Diehl et al., 2020; Ferreira et al., 2018; Kingsley et al., 2021; Siegner et al., 2018). More excitingly, indoor cultivation systems potentially offer more opportunities for the precise application of biofertilizers and agricultural microbiome harnessing (Bakker et al., 2012; Singh et al., 2020; Zhang et al., 2021).

The concept of biofertilizer was brought up against the chemical fertilizers which cause harmful impacts on living beings and the environment. Biofertilizer has been defined as living microorganisms that when applied to seeds, plants, or soil, colonize the rhizosphere or the interior of the plants and promote plant growth through diverse mechanisms (Mahanty et al., 2017). *Bacillus subtilis* (BS) showed great potential for being used as a biofertilizer as multiple beneficial effects of BS have been reported on crops including the improvement of nutrient availability, the alteration of phytohormone homeostasis, the production of antimicrobials, and the triggering of plant systematic resistance (Liu et al., 2022; Samaras et al., 2022). However, it has been noted that BS often fails to persist in the rhizosphere and thereby results in unstable and comprised functions when being applied in fields (Blake et al., 2021).

Here, we report a strategy promoting BS colonization in the rhizosphere of leafy green crops (*Lactuca sativa*) with the use of *Brassica rapa* subsp. Chinensis (Xiao Bai Cai) juice (XBCJ) and thereby achieving a synergistic promotion of leafy green vegetable production. A non‐target ultra‐performance liquid chromatography‐quadrupole time‐of‐flight mass spectrometry (UPLC‐Q‐TOF‐MS) analysis was employed to investigate the functioning components in XBCJ supporting BS growth. High‐throughput RNA‐based 16S rRNA gene sequencing and analysis were performed to depict the influence of BS and XBCJ over the global microbiome compositions in the plant rhizosphere.

*Brassica* leafy green vegetables are widely grown and consumed in the world, especially in Asian countries (Hong & Gruda, 2020). They are also one of the top‐grown leafy green vegetables on urban farms due to their high perishability and the concern of pesticide residuals when grown in the field (Łozowicka et al., 2012). Large quantities of roots, old leaves, as well as products approaching the expiration date are being generated. Since multiple bioactivities have been reported previously over *Brassica* leafy green vegetables (Chen et al., 2019; Favela‐González et al., 2020), it was of interest to explore sustainable options to close the food waste loop.

## EXPERIMENTAL PROCEDURES

### Bacteria culture

*Bacillus subtilis* subsp. *subtilis* (Ehrenberg) Cohn ATCC 21332, *Salmonella enterica* serovar Typhimurium ATCC 14028, and *Escherichia coli* ATCC 15597 were purchased from American Type Culture Collection and stored at −80°C. *B. subtilis* ATCC 21332 was activated by two continuous passages in Mueller Hinton broth (Oxoid, Basingstoke, Hampshire, England) at 37°C for 24 h. *S*. Typhimurium ATCC 14028 and *E. coli* ATCC 15597 were activated in tryptone soya broth (TSB; Oxoid) at 37°C for 24 h. Bacteria suspensions were washed twice in phosphate‐buffered saline (PBS; Vivantis, Selangor, Malaysia) to obtain the working cultures.

### Indole‐3‐acetic acid production measurement

Indole‐3‐acetic acid (IAA) production was tested with the Salkowski colorimetric assay, which was adapted from Gang et al. (2019). *B. subtilis* ATCC 21332 was cultured overnight in Luria‐Bertani broth, Miller (LB broth; Biobasic Asia Pacific, Singapore) with 0.15% l‐tryptophan (Sigma‐Aldrich, St. Louis, Missouri, United States) at 30°C, 120 rpm in an orbital shaking incubator under dark conditions, 1.2 mL aliquots of the bacteria culture were centrifuged (Centrifuge 5810 R, Eppendorf, Hamburg, Germany) at 10,000*×g* for 10 min at 4°C, 1 mL of the supernatant was mixed with an equal volume of Salkowski reagent (1 mL of 0.5 M FeCl_3_ [Fluka, Switzerland]) and 49 mL of 35% HClO_4_ (Merck, Rahway, New Jersey, United States). The mixture was then incubated at 30°C in a dark condition for 30 min and the absorbance at 536 nm was measured with a spectrophotometer (PowerWave XS2, Biotek, Winooski, Vermont, United States). For background correction, a blank was prepared using uninoculated LB broth with 0.15% l‐tryptophan in place of the bacteria culture supernatant. Finally, an IAA standard curve of 5–150 μg/mL (Sigma‐Aldrich) was used to determine the concentration of IAA produced.

### Preparation of XBCJ

*Brassica rapa* subsp. Chinensis (Xiao Bai Cai) was purchased from local markets. The vegetables were washed with tap water to remove the debris and then dried with a domestic salad spinner. Afterward, the vegetables were blended and stomached for 10 min. After standing by at room temperature for 1 h, the solid was removed with a cheesecloth. The filtered juice (XBCJ) was autoclaved at 121°C for 15 min before use.

### Effect of XBCJ on three bacteria in hydroponic nutrient solution

Hydroponic nutrient solution (solution A and solution B at a ratio of 1:1; Watercircle Hydroponics Pte Ltd.) was filter‐sterilized through 0.22 μm membrane filters (Sartorius Stedim Biotech, Göttingen, Germany), diluted by sterile deionized water to the electrical conductivity of 2000 μS/cm, and mixed with XBCJ at a ratio of 4:1. As a control, the hydroponic nutrient solution was mixed with sterile deionized water at 4:1. The washed bacteria working cultures were diluted by the hydroponic nutrient solution with or without XBCJ to a concentration of ~4 log CFU/mL and incubated at 37°C for 7 days. Aliquots of the culture were taken on Day 0 (immediately after preparation), Days 1, 3, 5, and 7 for microbiological analysis on Mueller Hinton agar (Oxoid) for *B. subtilis* and on tryptone soya agar (TSA; Oxoid) for *Salmonella* and *E. coli* with three biological replicates for each. The plates were incubated at 37°C for 24 h.

### UPLCQ‐TOF‐MS analysis

*Bacillus subtilis* ATCC 21332 cultured in hydroponic nutrient solution with XBCJ on Days 0, 1, and 3 as described above (6 biological replicates for each group) were centrifuged at 9000×*g* for 5 min (Eppendorf Centrifuge 5810 R, Hamburg, Germany) and the supernatants were filtered through 0.22 μm membrane filters (Sartorius Stedim Biotech). The resulting filtrates were frozen at −20°C and freeze‐dried (Buchi Lyovapor™ L‐300, Flawil, Switzerland). The resultants were dissolved in LC–MS grade water (Fisher Scientific, Hampton, New Hampshire, United States) at 20 mg/mL, and filtered through a 0.22 μm polytetrafluoroethylene (PTFE) filter. Quality control (QC) samples were prepared by mixing equal aliquots from each sample.

The analysis was performed with the Xevo G2‐XS QTOF MS system (Waters, Milford, Massachusetts, United States) with Luna C18 analytical column (250 × 4.6 mm, 100 Å) (Phenomenex, Torrance, California, United States) set at 30°C. The electrospray ionization (ESI) source was operated in negative ionization mode and mobile phases A and B were 0.1% formic acid in water and acetonitrile, respectively. The gradient elution was set to a flow rate of 0.7 mL/min and began with 10% B, which then increased to 100% B for 25 min, and 100% B was maintained for the next 9 min. The mobile phase was changed back to 10% B within 1 min before a post‐run of 5 min for column equilibration. The samples (3 μL each) were injected from the autosampler which operated at 8°C. The mass spectrometer parameters were as follows: capillary voltage and source temperature were set at 2.5 kV and 120°C, respectively. Nitrogen drying gas flow rate and temperature were set at 800 L/h at 500°C, respectively. The extracts were analysed in auto MS/MS mode (m/z range 100–1500) and data collection was done at five spectra per second in the extended dynamic range mode. The injection sequence began with 12 QC samples for repeatability, followed by the injection of a random sequence of samples to minimize signal drift effects, with a QC sample every 6 samples.

Raw LC‐QTOF‐MS analysis data files were processed in Progenesis QI (V.3.0, Waters), which performed retention time alignment (QC samples as reference) and 3D peak peaking based on intensity, m/z values, and retention time, with the parameters set as follows: all runs, chromatographic peak (minimum peak width set to 0.1 min), retention time (0–20 min), sensitivity (automatic, default) and limits (automatic). [M‐H]‐ and [M‐HCOO]‐ adducts were selected for deconvolution. The samples were categorized into 3 groups (Day 0, Day 1, and Day 3) according to the sample conditions, which were subjected to ‘between‐subject design’ comparison and one‐way analysis of variance (ANOVA) (*p* < 0.05) analysis. Metabolites that fulfilled three criteria (higher than 2‐fold change in intensity compared to Day 0 samples; average peaks are significantly different from Day 0 when tested with ANOVA at *p* = 0.05; confidence interval of less than 30%) were exported to EZinfo software (V.3.0.3, Umetrics, Umeå, Sweden) for multivariate data analyses.

Pareto scaling transformation was applied to the data processing before the principal component analysis (PCA) to reduce the effect of the metabolite magnitude (i.e., eliminate the masking effect). PCA was performed to determine the overall variations between samples, ensure QC clustering, and detect outliers. Meanwhile, orthogonal projection to latent structures‐discriminant analysis (OPLS‐DA) was performed to identify significant features between Day 1/Day 3, and Day 0 samples. Significant features with variable importance for prediction (VIP) of more than 2 were then selected and imported to Progenesis QI and Waters UNIFI software (V.2.0) for database identification. Identification was done based on their detected mass and mass spectrometric fragmentation patterns with the following databases: Food Database (http://foodb.ca/), Human Metabolome Database, MassBank (https://massbank.eu/MassBank/), National Institute of Standards and Technology Chemistry WebBook (https://webbook.nist.gov/chemistry/), National Library of Medicine, National Center for Biotechnology Information (https://pubmed.ncbi.nlm.nih.gov), Natural Product Atlas (https://www.npatlas.org) and Phenol‐Explorer (http://phenol-explorer.eu).

### Cultivation of lettuce

Seeds of lettuce (*Lactuca sativa*) were purchased from local farms. Before germination, lettuce seeds were disinfected with 70% ethanol for 1 min, followed by 1.2% ClO_2_ treatment for 12 min, and 3 times washed with sterilized deionized water. The seeds were then sowed in individual units of seeding starting garden trays. There were 40 units on each tray, and each unit was a frustum (3 cm × 3 cm square on top and 4 cm deep) filled with a peat substrate of 7 g/plant. Each tray was placed in a secondary container with 1 L of water (for the first 7 days) or 1 L hydroponic solution (with an electrical conductivity of 2000 μS/cm as described above) with or without BS and/or XBCJ supplement (from 7 to 21 days). The trays were kept in a plant growth climate chamber (FITOCLIMA 600, Aralab, Rio de Mouro, Portugal) with the cycling of 12 h of light at 350 μmol/m^2^/s and 12 h of darkness. The temperature and relative humidity were set at 25°C and 60%, respectively. The liquid levels in the secondary trays were filled up to the original levels with water every 2–3 days in compensation for the evaporation. On Day 7, the BS and XBCJ supplements were added to the secondary trays. *B. subtilis* ATCC 21332 was spiked at a level of ~8 log CFU/tray. XBCJ was with 20% of the volume whereas the electrical conductivity of the hydroponic solution was maintained at 2000 μS/cm consistently with all other groups.

### Microbiological analysis of the root blocks in the seeding starting garden trays

Currently, there is still some methodology inconsistency in plant rhizosphere microbiome studies (Brunel et al., 2020). In this study, the growth unit volume for each crop was rather small and by Day 21, the roots of lettuce developed across the whole units in the seeding starting garden trays. Therefore, on Day 21, after removing the edible parts of lettuce for quality analysis, the remaining roots together with the peat substrate (~10 g for each crop) were taken together as the ‘root blocks’, mixed with 10 mL buffered peptone water (BPW; Oxoid), and stomached for 2 min. *B. subtilis* was enumerated by plating the mixtures on *Bacillus cereus* selective agar (Oxoid), and the straw‐coloured colonies were counted as *B. subtilis* as described by the product manual.

For the microbiome sequencing, each root block was submerged in 30 mL autoclaved deionized water and vortexed until all the peat substrates were detached. Afterward, the samples were centrifuged at 8000×*g* for 10 min. The DNA was extracted from the pellets with FastDNA™ Spin Kit (MP Biomedical, France) with a 500 mg pellet as one sample following the manufacturer's instructions. The sequencing was completed on an Illumina NovaSeq 6000 system at NovogeneAIT Genomics Singapore Pte Ltd. The raw read files have been uploaded to NCBI Sequence Read Archive (SRA) with Accession No. PRJNA880783.

The data were processed with the open‐source Quantitative Insights into Microbial Ecology 2 (QIIME 2) pipeline (Bolyen et al., 2019). Sequences were denoised with the dada2 method and clustered into amplicon sequence variants (ASVs). The taxonomic classifier was trained using the Silva database (version 132). Taxonomies were assigned using feature‐classifier classify‐consensus‐blast.

### Quality analysis of lettuce crops

The physical parameters including height, weight, moisture content, texture, and colour were measured right after the lettuce samples were harvested. After being measured and weighed, three biological samples in each group were dried in an oven at 110°C overnight and the moisture content was calculated as the weight reduction before and after.

The texture measurement as the film burst strength (g) and the distance at burst (mm) was performed with TA.XT2i texture analyser (Stable Micro Systems, Surrey, UK). A 5 mm stainless steel spherical probe (P/5S) and a 5 kg load cell were used.

The colour of lettuce leaves was measured using a CM‐5 colorimeter (Konica Minolta Inc., Tokyo, Japan) in the CIELAB colour space. Three different areas (top, middle, bottom) of the outer leaves were measured, respectively, and the average readings of the three areas were taken as the result for one lettuce sample.

The antioxidant capacity analysis was performed as previously described (Huang et al., 2021). The lettuce samples were freeze‐dried and every 30 mg sample was extracted with 500 μL acetone‐water‐acetic acid (70:29.5:0.5 v/v) for 30 s vortex and 15 min sonication in an ice bath, followed by centrifugation at 20,000×*g* for 10 min. The above steps were repeated twice and the supernatant was combined for further analysis. For the total phenolic content (TPC) measurement, 100 μL of the sample extract was mixed with 200 μL of 10% Folin–Ciocalteu's phenol reagent and left at room temperature for 6 min. Afterward, 800 μL of 70 mM Na_2_CO_3_ was added and incubated at room temperature for 2 h in darkness. The absorbance of the mixture at 765 nm was with a microplate reader (PowerWave XS2, BioTek, Santa Clara, USA). The standard curve was developed with gallic acid with concentrations ranging from 0.016 to 0.25 g/L. The data were presented as mg gallic acid equivalent (GAE) per gram dry weight (mgGAE/gDW). For DPPH (2,2‐Diphenyl‐1‐Picrylhydrazyl) radical scavenging activity measurement, 20 μL of the sample extract was mixed with 180 μL of DPPH. The mixture was incubated for 2 h in darkness, and the absorbance of the mixture at 515 nm was measured. The DPPH quenched (%) was calculated based on the following formula:

$$\text{DPPH quenched}\;\left(\%\right)=\left[1-\left(\frac{A_{\text{sample}}-A_{\text{blank}}}{A_{\text{control}}-A_{\text{blank}}}\right)\right]\times 100\;\left(\%\right)$$

The calibration curve was prepared with Trolox with concentrations ranging from 50 to 500 μM. Data were expressed as μmol Trolox per gram DW (μmol TE/gDW).

### Statistical analysis

The SPSS 20.0 Statistics (IBM Corp., USA) was used to conduct the one‐way ANOVA analysis with the Tukey post hoc test. Significant differences were considered when *p* values were <0.05 (95% confidence intervals).

## RESULTS AND DISCUSSION

**FIGURE 1:**
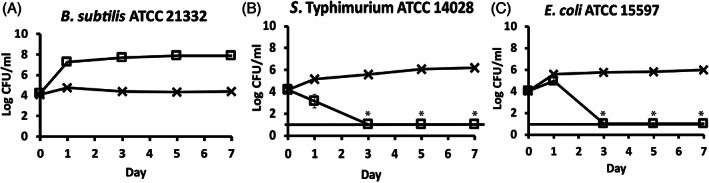
The fate of *Bacillus subtilis* ATCC 21332 **(**A) *S*. Typhimurium ATCC 14028 (B) and *Escherichia coli* ATCC 15597 (C) in hydroponic nutrient solution with (squares) and without (crosses) supplement of (Xiao Bai Cai) juice (XBCJ). Each data point represents the average of triplicates, and each error bar indicates the data range. *Below detection limit 1 log CFU/mL.

XBCJ showed opposite effects on *B. subtilis* and two *Enterobacteriaceae* bacteria strains in the hydroponic nutrient solution. The initiative of this study was inspired by the ad hoc observation of the differential effectiveness of XBCJ on *Bacillus* and two *Enterobacteriaceae* bacteria strains. The fate of *B. subtilis* ATCC 21332 was measured in hydroponic nutrient solution with and without supplement of XBCJ in comparison with *S*. Typhimurium ATCC 14028 and *E. coli* ATCC 15597. As a result, in hydroponic nutrient solutions without XBCJ, no significant change was observed for *B. subtilis* ATCC 21332 within 7 days (*p* < 0.05), and significant growth was observed for *S*. Typhimurium ATCC 14028 (2.04 ± 0.13 log CFU/mL increase from Day 0 to Day 7; *p* < 0.05) and *E. coli* ATCC 15597 (1.96 ± 0.22 log CFU/mL increase from Day 0 to Day 7; *p* < 0.05) (Figure 1). In contrast, with the presence of XBCJ, the population of *B. subtilis* ATCC 21332 quickly boosted from 4.22 ± 0.04 log CFU/mL to 7.27 ± 0.02 log CFU/mL within 1 day (*p* < 0.05) and maintained at the high levels till Day 7 (Figure 1A). The fate of *S*. Typhimurium ATCC 14028 and *E. coli* ATCC 15597 was the opposite of *B. subtilis* ATCC 21332, both dropped from ~4 log CFU/mL to levels below the detection limit (<1 log CFU/mL) within 3 days (Figure 1B, C).

From these results, we hypothesized that there are phytochemical contents in XBCJ showing antimicrobial effects towards *S*. Typhimurium ATCC 14028 and *E. coli* ATCC 15597, whereas *B. subtilis* ATCC 21332 was able to utilize some of the compounds in XBCJ for their growth. *B.subtilis* is a beneficial bacteria reported both as PRPG as well as with human health‐promoting potential (Liu et al., 2022; Permpoonpattana et al., 2012; Ritter et al., 2018; Samaras et al., 2022). *Salmonella*, on the other hand, is one of the most common human foodborne pathogens associated with fresh produce worldwide (Alegbeleye et al., 2018; Hanning et al., 2009). Previously, our group has isolated multiple *Salmonella* strains from hydroponic agriculture facilities and leafy green products (Tham et al., 2021) and has shown the capability of *Salmonella* to survive from contaminated seeds for an extended period in lettuce till harvest and pose serious cross‐contamination risks in the hydroponic farming environment (Li et al., 2022). Generic *E.coli* is often proposed as an indicator organism of fresh produce as its presence is related to (animal and/or human) faecal pollution (Uyttendaele et al., 2015). This differential effectiveness of XBCJ on different bacteria could be used to explore multiple application potentials. In this study, we focused on the support of XBCJ on *B. subtilis*, whereas the inhibition of XBCJ on *Salmonella* and *E.coli* was taken as controls to demonstrate the selectivity of XBCJ towards different bacteria.

### *Identification of components in XBCJ promoting* B. subtilis

**FIGURE 2:**
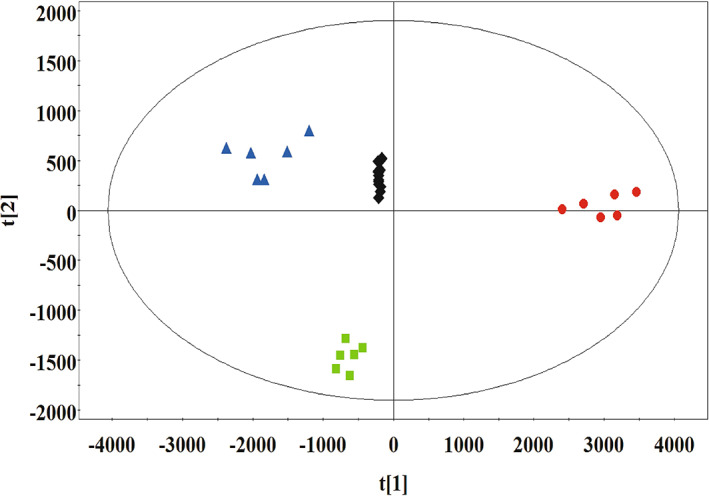
Multivariate statistical analysis (principal component analysis [PCA] plot) of compounds in (Xiao Bai Cai) juice (XBCJ) with *Bacillus subtilis* ATCC 21332 identified by ultra‐performance liquid chromatography‐quadrupole time‐of‐flight mass spectrometry (UPLC‐Q‐TOF‐MS). Six biological replicates were prepared and analysed for samples on Day 0 (red circles), Day 1 (green squares), and Day 3 (blue triangles). QC samples were prepared by mixing equal aliquots from each sample (black diamonds).

To identify the compounds in XBCJ supporting the growth of *B. subtilis* ATCC 21332, we compared the compounds in the systems of *B. subtilis* ATCC 21332 grown in hydroponic solution supplemented with XBCJ at different time points quantitatively. As shown in the PCA plot, the compounds in the systems on Days 0, 1, and 3 were differentiated clearly (Figure 2). Sixty‐three and 64 compounds were significantly down‐regulated (*p* < 0.05) in pairwise groups of Day 0 vs. Day 1 and Day 0 vs. Day 3 (Table S1). The top‐down‐regulated compounds with fold change > 10, VIP > 2, and *p* < 0.05 were listed in Table 1.

**TABLE 1:** Down regulated compounds in (Xiao Bai Cai) juice (XBCJ) with *B. subtilis* ATCC 21332 (fold change > 10, variable importance for prediction [VIP] > 2 and *p* < 0.05) Day 0 vs. Day 1 **(A)**, Day 0 vs. Day 3 **(B)** identified by ultra‐performance liquid chromatography‐quadrupole time‐of‐flight mass spectrometry (UPLC‐Q‐TOF‐MS) (negative ion mode).

Compound categories	Compounds	m/z	Retention time (min)	*p*‐Values	Fold change
(A)					
Phenolics	3‐O‐methyl quercetin	315.1	16.1	6.878E‐03	84.4
Phenolics	Caffeic acid	179.0	9.6	1.057E‐05	63.4
Phenolics	O‐acetyl vanillic acid	209.0	9.6	7.353E‐06	46.1
Phenolics	2‐oxo‐3‐(2,3,4,5‐tetrahydroxyphenyl) propanoic acid	227.0	6.0	4.581E‐08	24.2
Alcohol sugar	Sorbitol 6‐phosphate	261.0	12.0	1.779E‐11	24.8
Organic acids	Malic acid	133.0	4.3	1.973E‐04	23.0
Esters	Dimethyl sebacate	229.1	13.8	6.936E‐06	22.1
Long chain compounds	Nonylmalonic acid	229.1	13.8	6.936E‐06	22.1
Linoleic acids derivatives	(6E,8E,10R,12Z)‐10‐Hydroxy‐3‐oxo‐6,8,12‐octadecatrienoic acid	307.2	17.8	1.553E‐04	14.7
Oligopeptides	Glycyl‐phenylalanyl‐leucyl‐glycine	391.2	11.2	4.112E‐04	11.1
(B)					
Oligopeptides	Glycyl‐phenylalanyl‐leucyl‐glycine	391.2	11.2	4.112E‐04	426.4
Organic Acids	Malic acid	133.0	4.3	1.973E‐04	242.5
Esters	Dimethyl sebacate	229.1	13.8	6.936E‐06	138.7
Long chain compounds	Nonylmalonic acid	229.1	13.8	6.936E‐06	138.7
Phenolics	Caffeic acid	179.0	9.6	1.057E‐05	122.5
Linoleic acids derivatives	(6E,8E,10R,12Z)‐10‐hydroxy‐3‐oxo‐6,8,12‐octadecatrienoic acid	307.2	17.8	1.553E‐04	71.1
Phenolics	O‐acetyl vanillic acid	209.0	9.6	7.353E‐06	63.3
Phenolics	3‐O‐methyl quercetin	315.1	16.1	6.878E‐03	42.2
Phenolics	2‐oxo‐3‐(2,3,4,5‐tetrahydroxyphenyl) propanoic acid	227.0	6.0	4.581E‐08	26.0
Linoleic acids derivatives	9(S)‐HPODE	311.2	19.8	2.32E‐06	17.3
Hydroxy fatty acid	(10E)‐9‐Hydroxy‐12‐oxo‐10‐dodecenoic acid	227.1	13.9	1.65E‐08	15.2
Fatty acid	Traumatic acid	227.1	15.9	2.05E‐06	13.6

Noticeably, four phenolic compounds were consistently identified in the top down‐regulated compounds in both pairwise groups of Day 0 vs. Day 1 (out of the 10 compounds in Table 1A) and Day 0 vs. Day 3 (out of the 12 compounds in Table 1B). Indeed, multiple bacteria strains belonging to *Bacillus* spp. have been previously reported with the capabilities of degrading phenol compounds as their carbon source (Raj et al., 2007; Tam et al., 2006). On the other hand, phenolic compounds are an important class of phytochemicals with antimicrobial properties in many plants including *Brassica* vegetables (Favela‐González et al., 2020; Jaiswal et al., 2012; Vale et al., 2015). As reported previously, *Brassica rapa* subsp. Chinensis contains a wide range of phenolic compounds belonging to phenolic acids, flavonoids, and anthocyanins (Chen et al., 2019; Managa et al., 2020). Each phenolic compound is expected to have its unique spectrum of bacteria growth support and/or inhibition towards different strains. Therefore, the support of *B. subtilis* and the inhibition of *Salmonella* and *E.coli* of XBCJ as observed in this study is a concurrent effect of multiple compounds in the juice. The effect of XBCJ on *B. subtilis* with a unique microbiota background in plant rhizosphere is thus in need of comprehensive validation.

### The promotion of XBCJ in BS colonization in lettuce root blocks and the influence on the bacterial composition

Many strains belonging to *B. subtilis* have been reported with plant growth‐promoting effects due to multiple mechanisms (Blake et al., 2021). *B. subtilis* ATCC 21332 produces surfactant (Fox & Bala, 2000), which has been proven to be able to enhance the growth of plants (Buensanteai et al., 2008). Moreover, in this study, we found that *B. subtilis* ATCC 21332 was able to produce indole‐3‐acetic acid (IAA) (6.07 ± 1.39 μg/mL after 24 h cultivation and 11.2 ± 2.34 μg/mL after 48 h cultivation in broth from three independent measurements), which is comparable with the IAA producing bacteria as efficient biofertilizer inoculants to promote plant growth as previously reported (Mohite, 2013). Therefore, we used *B. subtilis* ATCC 21332 to demonstrate the promotion of XBCJ in BS colonization and the plant growth promotion accordingly.

**FIGURE 3:**
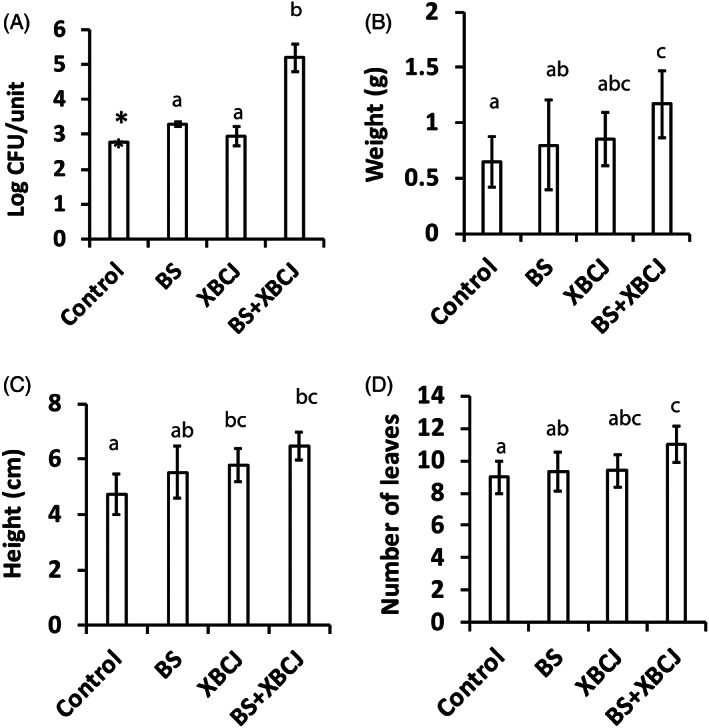
*Bacillus subtilis* counts (A), weight (B), height (C), and the number of leaves (D) of the edible parts of the lettuce crops with different treatments on Day 21. Each column represents the average of triplicates, and each error bar indicates the data range. *Below the detection limit. Different letters indicate significant differences (*p* < 0.05) between treatments.

Lettuce (*Lactuca sativa*) seeds germinated in seeding starting garden trays. After 7 days, freshly cultivated *B. subtilis* ATCC 21332 and freshly prepared XBCJ, separately or in combination, were mixed with the hydroponic nutrient solution and applied to the growth substrate of the lettuce crops. Two weeks later on Day 21, the *Bacillus* level was below the detection level (<2.77 log CFU/unit) from the roots together with the peat substrate in the control groups without BS/XBCJ supplement. Indeed, the spiked *B. subtilis* ATCC 21332 did not colonize well in the lettuce root blocks. *B. subtilis* population was enumerated at 3.30 ± 0.07 log CFU/unit from the lettuce root blocks in the BS group. In contrast, XBCJ facilitated *Bacillus* survival remarkably in the lettuce root blocks. In the BS + XBCJ group, *B. subtilis* was enumerated at 5.20 ± 0.39 log CFU/unit, being significantly higher than the *B. subtilis* enumerated in all other groups (*p* < 0.05, Figure 3A).

Accordingly, the lettuce crops from the BS + XBCJ group were significantly higher than the control group for all of the tested biomass‐related parameters (wet weight and length of the edible parts, numbers of leaves) (*p* < 0.05, Figure 3B–D). Whereas for the crops from the BS group and the XBCJ group, although the absolute values of plant biomass measurement were also higher than the control group, statistical significance was not observed (*p* > 0.05, Figure 3B–D).

For leafy green vegetable cultivation, our purpose of promoting plant growth is to accumulate higher biomass of the edible parts (shoots and leaves) and thus ensure higher agricultural yields. However, in the meantime, the organoleptic properties and nutritional values of the products cannot be compromised. In this study, no visual difference was noticed between the four groups except for the size of the crops (Figure S1). There was also no significant change in the texture, colour, and moisture contents of the crops between the four groups (*p* > 0.05; Table S2). Total phenolic contents and antioxidant activities, selected as the important nutritional parameters of the leafy green vegetables, were also tested to be comparable with and without the treatment of BS and/or XBCJ (*p* > 0.05; Table S2).

**FIGURE 4:**
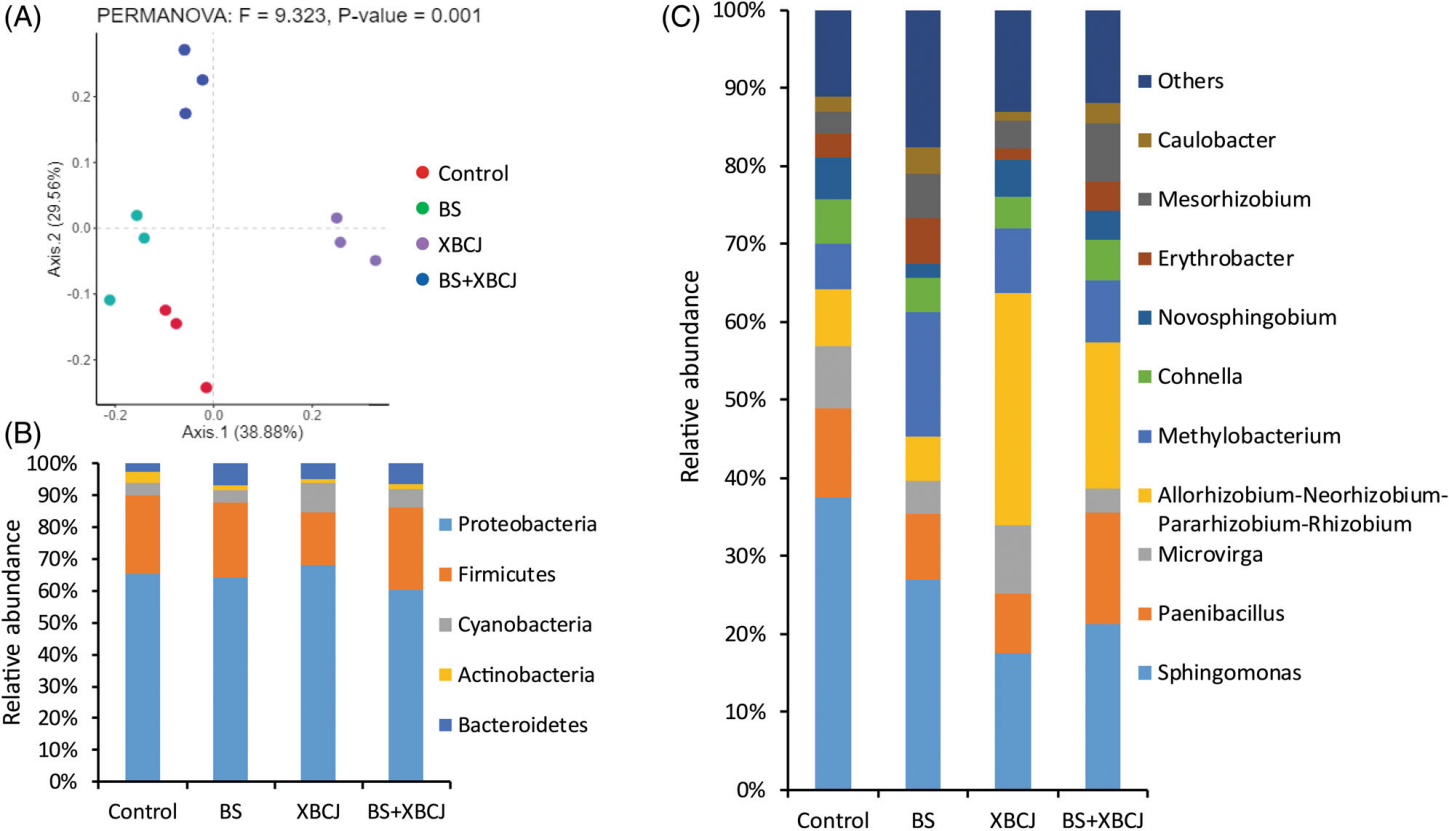
Taxonomy analysis of the microbiome in lettuce rhizosphere with different treatments at Day 21. Principal component analysis (PCA) at the OTU level revealed different diversities of samples from different treatments (A). The identified phyla (B) and genera (C) (average of three biological replicates for each treatment group) consist of >1% of the total microbiome in one or multiple samples.

As demonstrated above, the XBCJ components might act as antimicrobial agents and thus toxic for certain microorganisms (e.g., *S*. Typhimurium and *E. coli* as tested in this study) whereas can serve as the carbon source for some other microorganisms (e.g., *B. subtilis* as tested in this study) and thus promote their growth. When applied to the plants, it was expected that next to the promotion of BS colonization, XBCJ may also modify the microbiome composition of the root blocks, which was therefore analysed with the use of a high‐throughput RNA‐based 16S rRNA gene sequencing. As a result, no significance was observed in the α‐diversity (as demonstrated by the Shannon index and Chao1 using an OTU‐based analysis, Figure S2) between the four groups (*p* > 0.05). However, the β‐diversity analysis did reveal a clear differentiation of the microbial community structure between the four groups, especially the two groups with XBCJ (XBCJ and BS + XBCJ; Figure 4A). At the phylum level, XBCJ application significantly lowered the Firmicutes and enriched Cyanobacteria in comparison with the control and BS group (*p* < 0.05, Figure 4B), whereas when applied together with BS, the relative abundance of Firmicutes in the BS + XBCJ group was back to a higher level being comparable to the control and BS group (*p* > 0.05, Figure 4B). At the genus level, the XBCJ application significantly enriched the genera *Allorhizobium‐Neorhizobium‐Pararhizobium‐Rhizobium* (*p* < 0.05, Figure 4C), which are known to be diazotrophic (Purahong et al., 2022) and reported to be with bioremediation performance (Bai et al., 2020). As 16S rRNA gene sequencing only demonstrated the influence of XBCJ and BS treatment on the bacterial composition, the influence over fungi and other eukaryotic microorganisms remains to be investigated in the future.

## CONCLUSIONS

Since BS is not competitive in the plant rhizosphere microbiota, the promotion of BS requires a selective/elective strategy. In this study, the selective support of BS growth by XBCJ has been demonstrated with pure bacteria culture in a hydroponic nutrient solution and validated in a genuine scenario of lettuce cultivation. The chemical analysis together with the literature study suggested that the phenolic compounds could be the key class of phytochemicals being responsible for the selectivity, with some compounds serving as the carbon source for certain bacteria (including the spiked BS) whereas some compounds inhibiting certain accompanying microorganisms. In the scientific domain of human health, prebiotics is defined as ‘a substrate that is selectively utilized by host microorganisms conferring a health benefit’ (ISAPP, 2022). This study, for the first time to the best of our knowledge, brings up the concept of using the biowaste of *Brassica* vegetable production as the ‘prebiotics’ to promote the colonization of ‘probiotics’ in leafy green growth systems. The data accumulated in this study built up foundations for future studies to test and optimize more varieties of *Brassica* vegetable waste on a series of leafy greens, strengthen the effectiveness of biofertilizers, and harness the beneficial microbiome of urban farming systems.

## AUTHOR CONTRIBUTIONS

**Yingyue Li:** Conceptualization (supporting); data curation (equal); investigation (equal); methodology (equal); writing – original draft (equal). **Michelle Mei Zhen Ten:** Conceptualization (equal); data curation (equal); investigation (equal); writing – original draft (supporting). **Cliff An Ting Tham:** Conceptualization (supporting); methodology (supporting); resources (supporting). **Yan Xi Lim:** Data curation (equal). **Yuyun Lu:** Methodology (equal); validation (equal). **Dan Li:** Conceptualization (equal); funding acquisition (lead); project administration (lead); supervision (lead); validation (equal); writing – original draft (equal); writing – review and editing (lead).

## CONFLICT OF INTEREST STATEMENT

The authors declare no conflict of interest.

## Supporting information


**Figure S1.** The visual appearance of the lettuce crops in the four tested groups on Day 21.

**Figure S2.** α‐diversity as demonstrated by the Shannon index and Chao1 using an OTU‐based analysis of the high‐throughput RNA‐based 16S rRNA gene sequencing.

**Table S1.** Raw data from the non‐target ultra‐performance liquid chromatography‐quadrupole time‐of‐flight mass spectrometry (UPLC‐Q‐TOF‐MS).

**Table S2.** Quality and nutrition measurement of lettuce in different groups. Each data point represents the average of triplicates.

## Data Availability

All data are available to be requested from the corresponding author.
