# Supplementary figures and images for: Brassica rapa subsp. Chinensis juice enhances Bacillus subtilis selectively in leafy green production
Source: Environ Microbiol Rep. 2023 Mar 14;15(3):229–38. doi: 10.1111/1758-2229.13154 (PMC10464693; doi:10.1111/1758-2229.13154)

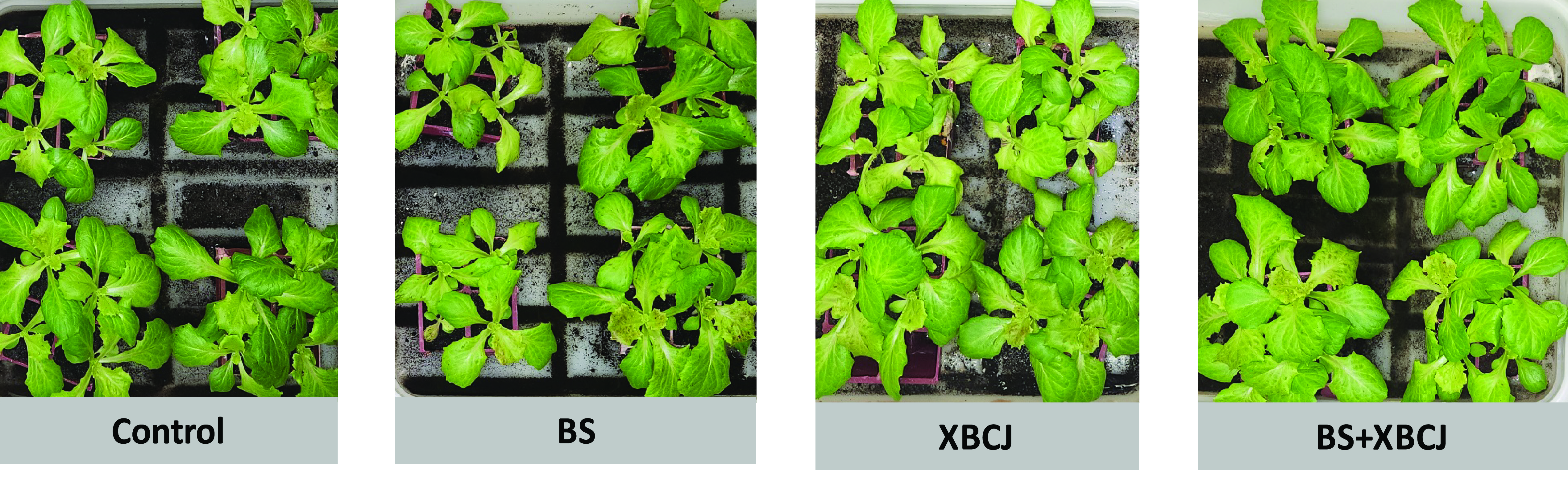

Supplement: Supplementary file 1 — Figure S1. The visual appearance of the lettuce crops in the four tested groups on Day 21. [file EMI4-15-229-s001.jpg]

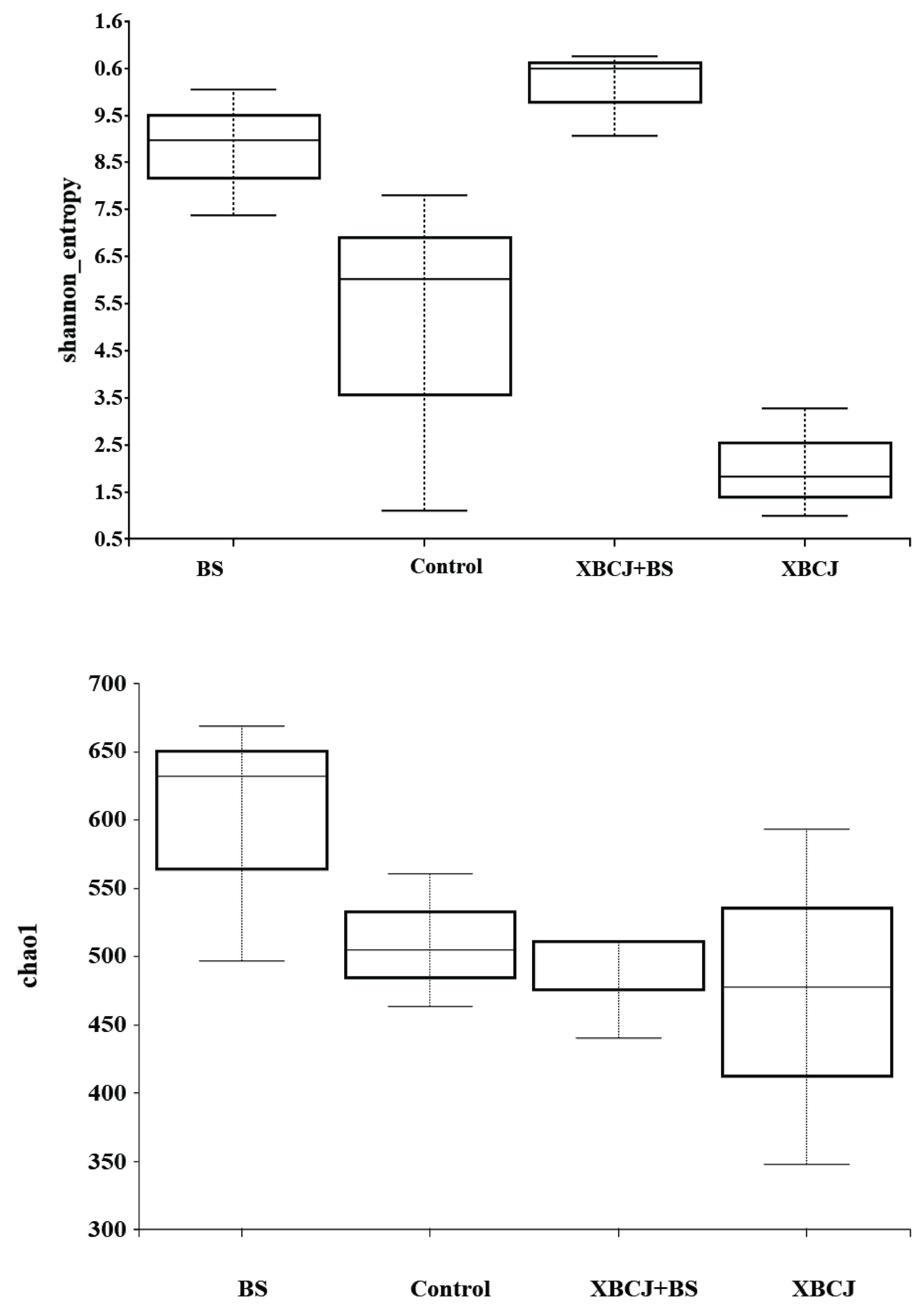

Supplement: Supplementary file 2 — Figure S2. α‐diversity as demonstrated by the Shannon index and Chao1 using an OTU‐based analysis of the high‐throughput RNA‐based 16S rRNA gene sequencing. [file EMI4-15-229-s002.jpg]
