# Supplementary material for: Brassica rapa subsp. Chinensis juice enhances Bacillus subtilis selectively in leafy green production
Source: Environ Microbiol Rep. 2023 Mar 14;15(3):229–38. doi: 10.1111/1758-2229.13154 (PMC10464693; doi:10.1111/1758-2229.13154)
Supplement: Supplementary file 4 — Table S2. Quality and nutrition measurement of lettuce in different groups. Each data point represents the average of triplicates. [file EMI4-15-229-s003.docx]

Table S2. Quality and nutrition measurement of lettuce in different groups. Each data point represents the average of triplicates.

|  | **Texture** | | **Colour** | | | **Moisture Content (%)** | **Total phenolic content (mgGAE/gDW)** | **DPPH**  **(µmol TE/gDW)** |
| --- | --- | --- | --- | --- | --- | --- | --- | --- |
|  | Film burst strength (g) | Distance at burst (mm) | L* | a* | b* |  |  |  |
| Control | 104.36 ± 8.73 | 2.96 ± 0.40 | 46.82 ± 2.90 | -10.31 ± 0.31 | 24.84 ± 2.25 | 95.22 ± 2.10 | 0.12 ± 0.01 | 253.37 ± 33.66 |
| BS | 85.29 ± 6.59 | 2.62 ± 0.19 | 48.51 ± 4.98 | -10.80 ± 0.36 | 28.54 ± 1.57 | 93.22 ± 0.53 | 0.10 ± 0.03 | 293.41 ± 80.01 |
| XBCJ | 95.37 ± 8.43 | 2.79 ± 0.31 | 51.28 ± 2.24 | -10.01 ± 2.77 | 30.65 ± 1.05 | 93.07 ± 1.20 | 0.11 ± 0.02 | 230.96 ± 65.14 |
| BS+XBCJ | 87.11± 13.63 | 2.82 ± 0.18 | 50.99 ± 1.87 | -10.59 ± 0.46 | 30.00 ± 0.96 | 94.09 ± 1.93 | 0.12 ± 0.01 | 266.64 ± 21.38 |
